# Wild Citrus CTV Genomic Data Provides Novel Insights into Its Global Transmission Dynamics

**DOI:** 10.3390/v17091162

**Published:** 2025-08-26

**Authors:** Xiang Li, Jun Zhou, Aijun Huang, Long Yi

**Affiliations:** 1 School of Life Science, Gannan Normal University, Ganzhou 341000, China; x18317513414@163.com (X.L.); 13102188352@163.com (J.Z.); hajgnnu@sina.com (A.H.); 2 National Navel Orange Engineering Research Center, Ganzhou 341000, China

**Keywords:** citrus tristeza virus, wild citrus, phylogeographic analysis, transmission dynamics, full-genome sequence

## Abstract

Citrus tristeza virus (CTV) is an important pathogen threatening the global citrus industry, but its evolution and transmission mechanism in wild citrus has not been clarified. Most of the existing studies are based on CTV-specific gene fragments, lacking genome-wide analysis. There is especially a lack of understanding of CTV transmission dynamics in wild citrus, which needs further investigation. In this study, wild citrus samples from three provinces of China were collected, virus genome data were obtained by high-throughput sequencing (HTS) technology and combined with public database data, and Bayesian phylogeographic inference was used to analyze virus composition characteristics in wild citrus, as well as the population genetic structure, temporal dynamic evolution, and spatial transmission mode of CTV. The results showed that Yunnan wild citrus samples contained the most abundant virus components, including CTV, Citrus Exocortis Viroid (CEVd), Citrus associated Ampelovirus 1 (CaAV-1), and Citrus Virus B (CiVB), while Jiangxi and Hunan samples only contained CTV and CEVd, with all samples showing mixed infection. Phylogenetic analysis showed that nine wild citrus CTV isolates were scattered in different evolutionary clades, and only 9.27% of genetic variation existed between the populations, while 90.72% of genetic variation existed within the populations, indicating little effect of geographic isolation on gene flow. The time to the most recent common ancestor (tMRCA) of CTV was estimated at 1360 CE, with subsequent divergence into two lineages, with population size stabilizing after a rapid increase in 1980–1990. Asia has been identified as the central source of CTV’s global spread, with key migration events including Asia to North America (1746), Asia to Oceania (1829), and Asia to South America (1965), coinciding with global maritime trade and the expansion of the citrus industry.

## 1. Introduction

Citrus is one of the most important cash crops in the world. China has been the primary producer for years [1]. Citrus plants, perennial woody species within the *Rutaceae* family, are believed to have originated in the southeastern foothills of the Himalayas, encompassing northeastern India, northern Myanmar, and northwestern Yunnan, China. As part of the Himalayan biodiversity hotspot, the mountainous regions of Yunnan are recognized as one of the world’s most floristically diverse areas [2]. The topography of peaks and deep valleys potentially act as barriers to species dispersal, while the region’s climatic, geological, and topographical diversity provides an ideal environment for floristic formation and development [3]. During long-term evolution and cultivation, citrus has accumulated various pathogens, including viruses and viroids, which severely impact yield and quality, causing significant economic losses.

Among these pathogens, CTV, a representative member of the *Closteroviridae* family, can weaken citrus trees and cause billions of USD in economic losses worldwide [4]. Aphids are the main vectors of CTV. While the molecular characteristics and pathogenic mechanisms of CTV, as a global disease, have been extensively studied, research on its evolutionary dynamics, particularly concerning whole-genome evolutionary forces and transmission patterns, remains limited. Whole-genome evolutionary dynamics have been investigated for other viruses like Tobacco mosaic virus, Tomato brown rugose fruit virus, and Tomato chlorosis virus [5,6]. Bayesian analysis of the *p25* gene by Wang et al. [7] indicated that CTV in China was primarily distributed in southern and coastal regions, with a substitution rate of 4.70 × 10^−4^ substitutions/site/year and a time to the most recent common ancestor (tMRCA) of around 1875 CE. This study confirmed that Chinese CTV originated from wild citrus in Hunan and Jiangxi, subsequently spreading to cultivated citrus areas in Sichuan, Chongqing, and Hubei, revealing its spatiotemporal transmission pattern. Bayesian analysis of the *p20* gene by Davino et al. [8] showed that the CTV introduced into Sicily, Italy, in 2002 comprised five evolutionary clades, with four co-circulating in the eastern citrus-growing area, while only the mild strain clade spread to remote areas after 2007, revealing a pattern of multiple introductions and complex migration. Benítez-Galeano et al. [9], based on Bayesian analysis of the *p25* gene, reported a substitution rate of 1.19 × 10^−3^ substitutions/site/year for the CTV-NC lineage, with a tMRCA of around 1977. This study demonstrated that CTV-NC originated in the USA and subsequently spread globally to Argentina, Uruguay, Brazil, China, etc., revealing its spatiotemporal transmission pattern mediated by infected nursery stock transport and aphid vectors. However, these studies focused on specific CTV gene fragments for phylogenetic analysis and did not conduct Bayesian evolutionary analysis on the whole CTV genome.

Wild citrus species, as close relatives of cultivated varieties, are considered “natural reservoirs” for long-term virus coexistence [10]. Virus transmission between wild and cultivated citrus can occur via insect vectors or natural grafting [11,12]. However, current research on viral populations in wild citrus is often limited to single viruses or localized regions, lacking systematic comparative analysis. For instance, whether genotype distribution in wild hosts exhibits geographical specificity, or whether viral population diversity is driven by ecological factors (e.g., altitude, host diversity), remains largely unanswered [13]. Therefore, this study collected wild citrus from three provinces in China and conducted HTS to supplement and enrich the genomic data of CTV. Combined with the existing data in the NCBI database, the population dynamics of wild citrus CTV were systematically analyzed, aiming to provide a theoretical basis for virus tracing and control.

## 2. Materials and Methods

### 2.1. Sample Collection and Processing

From 2019 to 2024, a total of 103 wild citrus samples were collected across three provinces in China. For each specimen, morphological characteristics were documented, geographic coordinates were recorded using GPS, and digital photographs were archived for reference. Fresh leaf tissues were immediately flash-frozen in liquid nitrogen and subsequently stored at −80 °C in an ultra-low-temperature freezer until RNA extraction. Based on a representative sampling strategy, 3–4 samples per geographical region were selected for HTS, resulting in a final selection of 11 samples, which were ultimately chosen for viral discovery and composition analysis. The sequenced samples originated from the following locations: Jiangyong County, Hunan Province: collected in 2019 (n = 4; sample IDs: JY1-JY4); Chongyi County, Jiangxi Province: collected in 2024 (n = 3; sample IDs: NDX45, NDX50, NDX84); Ailao Mountains, Yunnan Province: collected in 2024 (n = 4; sample IDs: YX11, YX17, YX22, YX26).

### 2.2. RNA Extraction, Library Preparation, and Sequencing

Frozen leaf samples stored at −80 °C were ground to a powder in liquid nitrogen. Total RNA was extracted from 100 mg tissue using TRIzol reagent (TIANGEN, Beijing, China), according to the manufacturer’s instructions. RNA concentration and purity were assessed using a NanoDrop 2000 spectrophotometer (Thermo Fisher, Shanghai, China). Samples with A260/A280 ratios between 1.7 and 2.5 and concentrations of ≥100 ng/μL were retained. RNA integrity (RIN ≥ 7.0) was further verified by 1% agarose gel electrophoresis and an Agilent 2100 Bioanalyzer (Agilent Technologies, Santa Clara, CA, USA). Ribosomal RNA was removed using the Ribo-off rRNA Depletion Kit (Vazyme, Nanjing, China). Sequencing libraries were constructed using the NEBNext Ultra II RNA Library Prep Kit (NEB, Ipswich, MA, USA). Library concentration was quantified using a Qubit 2.0 Fluorometer (Thermo Fisher Scientific, Shanghai, China), and the insert size (250–350 bp) was confirmed using the Agilent 2100. Finally, paired-end sequencing (2 × 150 bp) was performed on an Illumina NovaSeq 6000 platform (Novogene, Beijing, China).

### 2.3. Sequence Assembly and Virus Discovery

Raw sequencing reads were preprocessed using Trimmomatic v0.39 [14] with parameters set to SLIDINGWINDOW:4:20, LEADING:20, TRAILING:20, and MINLEN:50 to remove low-quality sequences and adapter contamination. Cleaned reads were mapped to the sweet orange reference genome (*Citrus sinensis* v2.0, NCBI Assembly GCF_000317415.1) using HISAT2 v2.2.1 [15] with default parameters. Host-derived sequences were filtered out. Unmapped reads were de novo assembled using Trinity v2.13.2 [16] with a minimum contig length of 200 bp. Contigs were annotated by BLASTn comparison against the NCBI viral reference database, using an E-value threshold < 1 × 10^−6^ and coverage ≥ 70%.

### 2.4. Viral Composition Identification in Wild Citrus and CTV Phylogenetic Analysis

Viral species composition in wild citrus samples was identified by counting the number of valid reads mapped to known viral reference genomes. For viruses identified by HTS, specific primers (Table S1) were validated using reverse transcription PCR (RT-PCR). Briefly, total RNA was extracted from samples, cDNA was synthesized using a reverse transcription kit, PCR amplification was performed using cDNA as a template, amplification products were detected by agarose gel electrophoresis, and virus identification was confirmed by the presence or absence of target bands.

Based on annotation results, CTV-related sequences were assembled using SeqMan7.1.0.44 software. Nine near-full-length CTV genome sequences were successfully obtained from the 11 sequenced samples. These were combined with representative CTV isolate sequences from the NCBI nucleotide database. Multiple sequence alignment was performed using MAFFT7.526 software to identify conserved regions and variable sites. To detect potential recombination events, a comprehensive analysis was conducted using seven methods (RDP, GENECONV, BOOTSCAN, MAXCHI, CHIMAERA, SISCAN, and 3SEQ) within the RDP v4.95 software [17]. To reduce false positives, recombination events were considered statistically significant only if supported by at least four methods. Based on the alignment, a maximum likelihood (ML) phylogenetic tree was constructed using IQ-TREE1.6.12 software [18]. Branch support was assessed with 1000 bootstrap replicates to clarify the evolutionary relationships between the CTV sequences obtained in this study and the CTV sequences of different geographical origins and genotypes globally. The newly obtained CTV whole-genome sequences have been deposited in the GenBank database (accession numbers provided in the results).

### 2.5. CTV Population Genetic Differentiation Analysis

For population genetic differentiation and phylogeographic analysis of CTV, 117 near-full-length CTV genome sequences from China, Spain, USA, Iran, Croatia, Angola, Montenegro, India, France, and South Africa, spanning 1992–2023, were retrieved from the NCBI nucleotide database (http://www.ncbi.nlm.nih.gov), accessed on 21 March 2025. These sequences, accompanied by their collection dates and geographical origins (Table S2), provide critical temporal and spatial metadata for Bayesian phylogenetic analyses, supporting inferences of evolutionary dynamics and spatial transmission patterns. These retrieved sequences were integrated with the nine newly sequenced CTV sequences from this study, creating a dataset of 126 sequences for subsequent analysis. Based on geographical distribution, the 126 CTV sequences were divided into six populations corresponding to Europe, Asia, North America, Africa, South America, and Oceania. *Fst* values (Fixation index), a classical parameter measuring the degree of genetic differentiation between populations, were calculated using Arlequin v3.5 software [19]. Interpretation followed established standards [20] as follows: *Fst* values of 0.05–0.15 indicate moderate differentiation; 0.15–0.25 indicate high differentiation; and >0.25 indicate very high differentiation. Furthermore, *Fst* values can assess gene flow levels, where *Fst* < 0.33 generally suggests frequent gene flow between populations, while *Fst* > 0.33 indicates relatively restricted gene flow.

### 2.6. CTV Temporal Dynamics and Molecular Clock Analysis

To analyze the temporal evolutionary characteristics and population history of CTV, Bayesian time-scaled tree construction and population dynamic analysis were performed on the 126 full-length genome sequences. The substitution model was selected using ModelFinder [21] within IQ-TREE [18] based on the Bayesian Information Criterion. Time-scaled tree inference was implemented in BEAST v2.6 [22] using the Markov Chain Monte Carlo (MCMC) approach for molecular clock analysis. A Jukes–Cantor-corrected UPGMA tree was used as the starting tree for MCMC, providing an initial topological framework. The HKY nucleotide substitution model was selected, with the transition/transversion rate ratio parameter (*κ*) set to 2.0 to account for base substitution heterogeneity. The uncorrelated lognormal relaxed clock model was used for branch rates, with the global rate mean set to 0.001 substitutions/site/year and a standard deviation of 0.3333, allowing for stochastic rate variation among the branches to accommodate potential rate heterogeneity in virus evolution. The GMRF SkyGrid model [23] was used as a coalescent prior to model changes in effective population size over time; moreover, this model flexibly fits historical changes in effective population size using a piecewise constant function, suitable for capturing complex population expansion or contraction dynamics.

For optimal time tree extraction, the MCMC tree set was processed using TreeAnnotator v2.6. To address ambiguous root placement suggested by high branches near the root node under default parameters, the parameters were optimized to enhance tree structure reliability as follows: a 30% burn-in was set to exclude the effects of initial non-stationary iterations; the HIPSTR algorithm was enabled to handle polytomies; and the root node was constrained by specifying common ancestor heights. The maximum clade credibility tree was finally extracted.

Population dynamic analysis utilized the EBSP method. The extended Bayesian skyline plot served as a flexible tree prior, enabling the inference of temporal changes in effective population size [24]. Posterior estimates (median and 95% Highest Posterior Density interval, HPD) of the effective population size at various time points were extracted from the EBSP file output by BEAST using Tracer v1.7. The results were visualized using the ggplot2 package [25] in R, plotting the median curve with time on the x-axis and effective population size on the y-axis, with the 95% HPD interval shaded, to intuitively present CTV population dynamics over time. This analysis pipeline, through precise molecular evolutionary model settings, tree structure optimization, and population dynamics visualization, provided quantitative phylogenetic and population genetic evidence for revealing CTV’s spatiotemporal transmission patterns. Additionally, phylogeographic analysis was performed using SpreaD3 v3 [26] with default parameters to investigate CTV transmission pathways between different geographical regions. For efficient processing of phylogenetic tree data, the treeio package [27] was utilized for convenient input/output and rich annotation of trees, tightly integrating with the overall workflow.

### 2.7. CTV Phylogeographic Diffusion and Population Dynamic Analysis

To gain deeper insights into CTV dissemination, spatial transmission patterns were reconstructed using Bayesian phylogeographic inference implemented in BEAST 10.5.0-BETA5 software. The six geographical regions were encoded as discrete states, and posterior distributions of parameters were estimated via MCMC sampling, as described above. MCMC simulations were run for 100 million steps across three independent chains, sampling every 10,000 steps. The first 10% of iterations were discarded as burn-in. Convergence was assessed using Tracer v1.71, ensuring all parameters had effective sample sizes (ESS) > 200. The best-supported pairwise diffusion routes were identified using Bayes Factors (BF) in SPREAD3 [28]. Migration routes were considered statistically significant if BF > 3 and the mean posterior probability was > 0.5 [5]. The expected number of location state transitions along branches [29] was also estimated.

## 3. Results

### 3.1. Sampling and Sequencing

**Figure 1:**
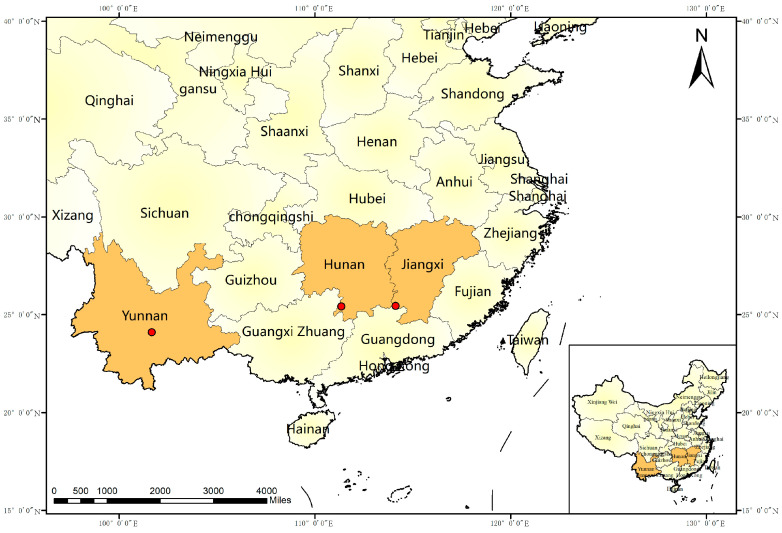
Geographic distribution of wild citrus CTV sampling points. The orange marks the sampling locations in three Chinese provinces, and the sampling time is from 2019 to 2024. Red dots: sampling locations.

Between 2019 and 2024, 103 wild citrus samples suspected of CTV infection were collected from three provinces in China (Figure 1). Following RNA extraction and concentration screening, 11 samples underwent HTS. Sequencing data showed raw data volumes of 12–26 GB per sample. After quality filtering, the number of valid reads ranged from 72 to 85 million for Yunnan samples, 42 to 53 million for Jiangxi samples, and 164 million for Hunan samples (Table 1). Based on contig annotation and assembly using SeqMan software, nine near-full-length CTV genomes were successfully obtained from the 11 sequenced samples, originating from the following: Jiangyong and Dao Counties, Hunan (JY1-JY4); Chongyi County, Jiangxi (NDX45, NDX50); and Ailao Mountains, Yunnan (YX11, YX17, YX26). Additionally, 117 near-full-length CTV sequences from China, Spain, USA, Iran, Croatia, Angola, Montenegro, India, France, and South Africa (1992–2023) were retrieved from the NCBI nucleotide database. These were combined with the newly sequenced data to form a dataset of 126 sequences for subsequent analysis.

### 3.2. Viral Composition in Wild Citrus and Phylogenetic Analysis

Virus identification based on HTS revealed four viruses in the 11 wild citrus samples: CTV, Citrus Exocortis Viroid (CEVd), Citrus-associated Ampelovirus 1 (CaAV-1), and Citrus Virus B (CiVB). Significant regional differences in viral species composition were observed (Table 2). All four viruses were detected in the Yunnan samples (YX11, YX17, YX22, YX26), indicating the richest viral composition. Only CTV and CEVd were detected in the samples from Jiangxi (NDX45, NDX50, NDX84) and Hunan (JY1-JY4). The RT-PCR validation results were consistent with HTS, confirming the accuracy of virus identification across the regions.

**Figure 2:**
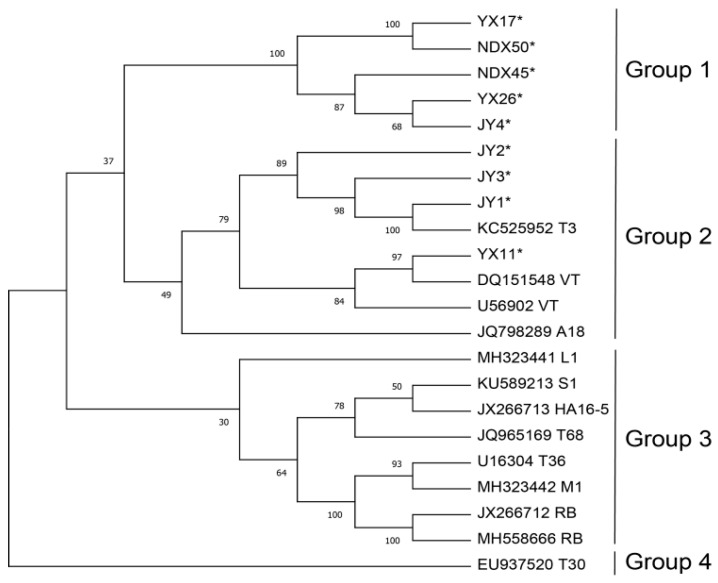
Maximum likelihood phylogenetic tree based on CTV genome sequence. The figure includes 9 wild isolates (marked with * in this study) and global representative strains. The phylogenetic support rate was obtained by 1000 bootstrap tests.

A maximum likelihood phylogenetic tree based on the nine near-full-length CTV genome sequences obtained in this study and the representative global isolates, using an outgroup based on CTV gene sequences, are shown in Figure 2. In the tree topology, the nine CTV isolates from wild citrus were clustered into different branches. Isolates YX17, YX26, NDX45, NDX50, and JY4 clustered into Group 1; JY1, JY2, JY3, and YX11 clustered with *T3*, *VT*, and A18 into Group 2; *LA*, *RB*, *MA*, *T36*, *T68*, *S1*, *T30*, and *HA16-5* clustered into Group 3; and the *T30* isolate formed a separate Group 4, positioned as the outermost group. Notably, isolates from the same region (Yunnan YX11/YX17/YX26, Jiangxi NDX45/NDX50, Hunan JY1/JY2/JY3/JY4) did not form monophyletic clusters.

### 3.3. Genetic Differentiation Analysis of CTV Populations

The nine CTV genome sequences obtained in this study have been deposited in GenBank (Accession numbers: SRR34552294, SRR34552612, SRR34552644, SRR34553224, SRR34553347, SRR34553363, SRR34554392, SRR34554391, SRR34554446, SRR34554472-SRR34554474). Analysis of Molecular Variance (AMOVA) based on the 126 sequences (Table S3) revealed that genetic variation among the CTV populations accounted for only 9.27% (*Fst* = 0.093, *p* < 0.001), while 90.72% of the variation resided within the populations. The gene flow parameter *Nm* = 2.444 indicated low genetic differentiation among the CTV populations across continents but high genetic diversity within the populations and frequent gene flow.

### 3.4. Temporal Dynamics of CTV

**Figure 3:**
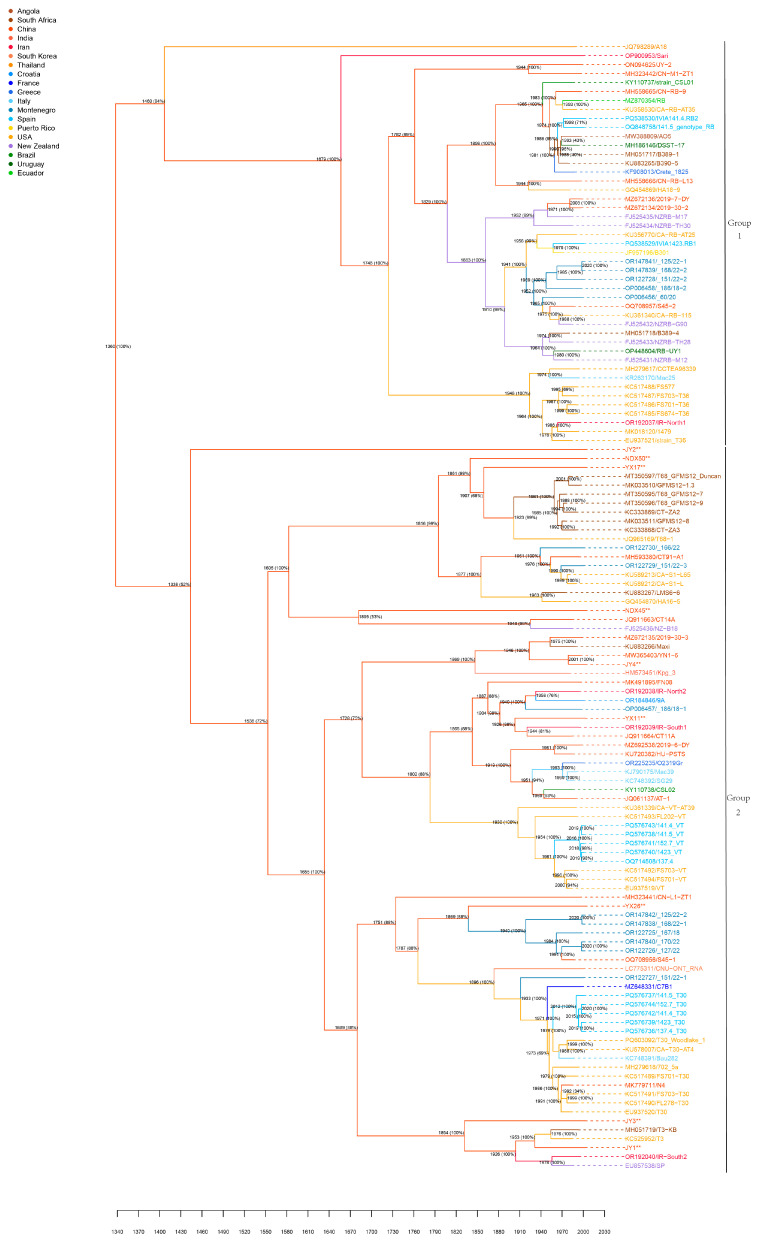
Time-scaled Bayesian MCC phylogenetic tree of CTV based on 126 full-length genome sequences. Among the isolates, the 9 wild citrus ones obtained in the present study are marked as ** in the diagram. The branch color corresponds to the geographical lineage and the time axis unit is CE.

MCMC runs for the BEAST analysis showed good convergence, with all parameters having ESS > 200. The maximum clade credibility tree from Bayesian phylogenetic analysis (Figure 3) revealed that the tMRCA of CTV dates back to 1360 CE (95% HPD: 715 Before Common Era (BCE)–1802 Common Era (CE)). CTV diverged into two major lineages: Lineage 1, predominantly in South America and Oceania, which diverged around 1460 CE (84% HPD: 901–1849 CE), with the earliest sequence sampled in 1992; and Lineage 2, predominantly in Asia, Europe, Africa, and North America, which diverged around 1338 CE (52% HPD: 988–1627 CE), with the earliest sequence sampled in 1996. The newly sequenced isolates (JY/NDX/YX) were all assigned to Lineage 2 but were distributed across distinct subclades within this lineage. This suggests that, while strains from Yunnan, Hunan, and Jiangxi share a relatively recent common ancestor, significant local differentiation occurred during subsequent evolution, likely due to geographical isolation, differing agro-ecological environments, or host differences.

### 3.5. Migration Events and Population Dynamic History of CTV

**Figure 4:**
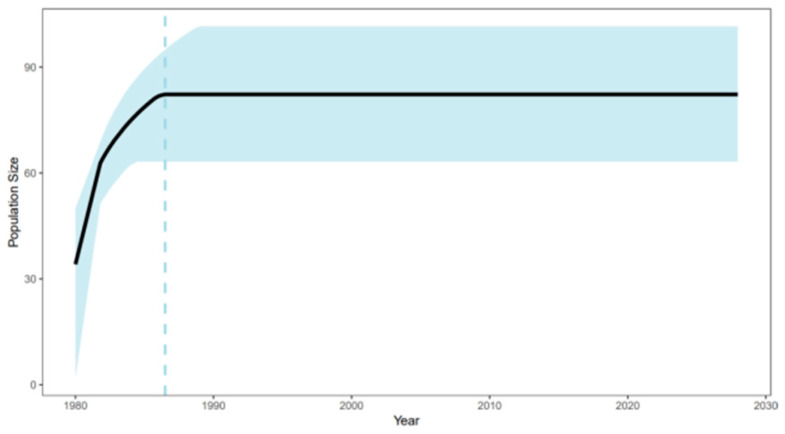
Historical dynamic changes in effective population size of CTV (EBSP analysis). The black line represents the median estimate, the light blue shadow represents the 95% confidence interval, and the horizontal axis is the CE year.

Reconstruction of population size history using EBSP (Figure 4) revealed multiple changes in the CTV population size over time. Between 1980 and 1990, the CTV population size showed rapid growth, followed by relative stabilization after 1990. The light blue shaded area represents the 95% confidence interval for the population size estimate, reflecting analytical uncertainty. This result indicates that CTV experienced a phase of rapid dissemination in its early history, later entering a relatively stable phase, possibly due to environmental or host-related factors.

**Figure 5:**
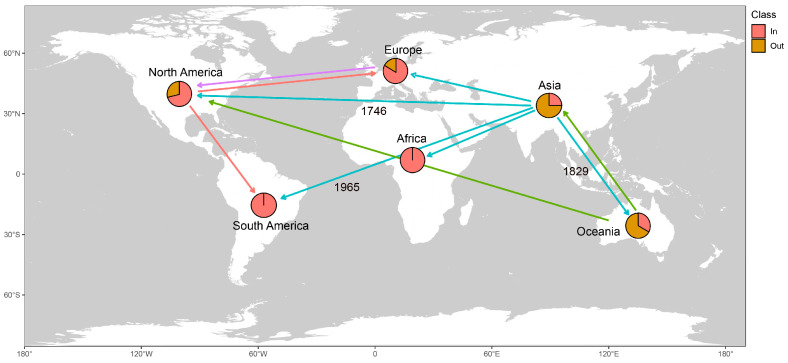
Spatiotemporal diffusion network of CTV global transmission routes. Arrow colors indicate different transmission routes, and key events are annotated with the years of migration.

Phylogeographic analysis (Figure 5) identified ten significant CTV migration routes: Asia to Africa, Europe, North America, South America, Oceania; North America to Europe, North America; Oceania to Asia, North America; and Europe to North America. Key migration events included the following: CTV dispersal from Asia to North America in 1746 CE, from Asia to Oceania in 1829 CE, and from Asia to South America in 1965 CE. The maximum clade credibility (MCC) tree and diffusion trajectory map supported these results, with Asia showing significantly higher outward migration frequency than other regions.

## 4. Discussion

This study focused on wild citrus as the primary research subject. Through integrated whole-genome sequencing, phylogenetic analysis, population genetic differentiation, and Bayesian phylogenetic analysis, the global transmission dynamics and evolution rules of CTV were analyzed from the perspective of wild hosts; moreover, this provides novel insights into the cross-regional transmission mechanism of CTV through multi-level evidence verification.

As natural hosts free from artificial selection pressure, wild citrus exhibited significant regional variation in viral composition, closely linked to geographical and ecological factors. The Yunnan mountainous area has become one of the regions with the richest plant diversity due to having complex terrain and climatic conditions [2], which may be an important reason for the detection of CTV, CEVd, CaAV-1, and CiVB in samples from Ailao Mountain, Yunnan Province. In lowland hilly areas of Jiangxi and Hunan, the host simplification caused by agricultural activities limited the expansion of virus lineage, only CTV and CEVd were detected, and mixed infection of these two viruses existed in all samples of Jiangxi and Hunan. Wild citrus, as a close relative of cultivated varieties, is considered a “natural reservoir” for long-term virus coexistence [10]. However, the phenomenon of multiple virus coexistence in Yunnan samples in this study provides a natural research model for exploring the interaction mechanism between viruses and also confirms the possibility of virus transmission between wild citrus mediated by vector insects or natural grafting [11,12].

Phylogenetic analysis combined with temporal dynamic modeling revealed the underlying evolutionary dynamics of CTV. The phylogenetic tree showed that nine wild citrus CTV isolates were scattered in different branches, and isolates from the same area did not form monophyletic groups. The *Fst* value was only 9.27%, indicating that genetic variation among populations was mainly due to geographical isolation, while genetic variation within populations was as high as 90.72%. Together, these results suggest that gene flow across regions (*Nm* = 2.44) is a major factor in offsetting the effects of geographic isolation. This is consistent with the results of low differentiation based on *p25* gene fragment analysis by [7], suggesting that the virus still maintains certain gene exchange during transcontinental transmission, which may be related to long-distance transmission promoted by global trade and human mobility [9]. The temporal tree further gives this conclusion a temporal dimension. All wild citrus isolates belong to lineages dominated by Asia, Europe, Africa, and North America, and this lineage differentiation dates back to 1338, much earlier than the sampling time of existing sequences after 1992. This characteristic of “ancient differentiation combined with continuous gene flow” supports the hypothesis that wild citrus acts as a “reservoir” for the long-term evolution of CTV [10], revealing the dynamic equilibrium of viruses under the joint action of natural and human factors, such as seedling trade and vector migration. Geographic isolation, such as that caused by the high mountains and deep valleys of Ailao Mountain, promotes local differentiation, while cross-regional transmission, such as that caused by trade exchanges between Asia and America, maintains gene exchange, and finally forms a unique topology of “scattered clusters” in the phylogenetic tree. This is consistent with previous studies on wild citrus CTV based on the coat protein gene [30] or *p23* gene [31] for wild citrus CTV, and Martin et al. [32] for cultivated citrus CTV, further indicating that gene flow and gene recombination may exist among CTV isolates from different geographical origins.

The contribution to genome-wide Bayesian analysis was to integrate 126 global sequences to trace CTV’s most recent common ancestor (tMRCA) back to 1360 (95% HPD: 715 BCE-1802 CE), a timescale that far exceeded estimates based on specific gene segments. For example, Wang et al. [7] inferred the tMRCA of China CTV to be around 1875 based on *p25* genes, while Benítez-Galeano et al. [9], with *p25* gene analysis for CTV-NC lineages, showed its tMRCA to be around 1977. This significant difference underscores the advantage of genome-wide analysis for reconstructing deep evolutionary histories [2]. Analyses based on specific gene segments can be biased by strong selective pressures, recombination events, or rate heterogeneity [33], whereas genome-wide data, encompassing more neutral or near-neutral sites, provide a more comprehensive capture of viral evolutionary signals [34,35]. Lineage 1, including South America and Oceania, and Lineage 2, including Asia, Africa, and Europe, diverged around 1460 and 1338, which is highly consistent with ecological isolation prior to the rise of medieval global maritime trade [36]. This suggests that early natural transmission may have been the driving force for initial differentiation, while modern colonial expansion and globalization of citrus industries accelerated the convergence of transcontinental lineages [37]. Although the newly detected sequences, JY, NDX, and YX, in this study are in Lineage 2, they form independent small branches, indicating that the Yunnan, Hunan, and Jiangxi strains all evolved from a relatively close common ancestor; however, significant local differentiation occurred in subsequent evolution, which may be related to geographical isolation, differences in agricultural ecological environment, and host adaptation evolution [38].

Phylogeographic analysis further quantified the global transmission path of CTV, confirming that Asia was the central source of virus spread and that key migration events of Asia → North America (1746), Asia → Oceania (1829), and Asia → South America (1965) were highly consistent with critical periods of global maritime trade expansion and citrus industry development [36]. In particular, the spread from Asia to North America (1746) occurred during a period of active transatlantic trade during the colonial period, while the spread to South America (1965) coincided with a period of accelerated modern globalization and a surge in international agricultural trade [37]. This finding aligns with the population dynamics inferred from the EBSP analysis. The CTV population’s exponential growth from 1980 to 1990 coincided with global citrus intensification periods, such as large-scale introduction in China [39], while the stabilization period after 1990 may have resulted from the introduction of resistant rootstocks [40] and the intensification of quarantine measures [41]. This Asia-centric multidirectional spread pattern is similar to the global transmission characteristics of other plant viruses studied by phylogenetic geography, such as tomato yellow leaf curl virus (TYLCV) [42], highlighting the central role of international trade in virus globalization. This knowledge provides a key anchor point for developing targeted control strategies, such as enhancing virus screening in citrus nursery stock exported from Asia to effectively disrupt core transmission pathways.

## 5. Conclusions

Utilizing wild citrus as a unique study system, this research integrated geographical variation in viral composition, spatiotemporal evolutionary patterns, and the historical drivers of global spread into a unified framework through genome-wide multidimensional analysis. This study provides genome-wide support for the view that wild citrus may have been a natural reservoir of long-term evolution of CTV, but also delineates a continuum of evidence, extending from static phylogenetic relationships to dynamic historical processes, elucidating the mechanisms of cross-continental transmission in plant viruses through high-precision analysis of genome-wide data, and has provided a scientific basis for virus risk management in the global citrus industry. The temporal focus of the analyzed sequences, primarily post-1992, may limit the accuracy of reconstructing older CTV evolutionary events. Future studies should expand spatiotemporal sampling by targeting viral sequences from older archived specimens and incorporating samples from undersampled regions (e.g., Africa, Central Asia). This would enable a more precise estimation of the tMRCA and reconstruction of comprehensive migration routes. Secondly, we can combine the key differentiation lineages found in this study to study the pathogenicity differences and interaction mechanisms with different hosts.

## Figures and Tables

**Table 1:** Summary of HTS data for wild citrus samples. The table contains parameters such as sampling sites, sample numbers, data volume, and quality assessment.

Sampling Locations	Sample	Bases (GB)	Total Reads	Mapping Reads	Q30 (%)	Avg. Quality
Chongyi	NDX45	9.442	59,698,544	42,356,972 (70.95%)	94.97	38.955
	NDX50	8.208	52,472,188	46,027,772 (87.72%)	95.57	39.085
	NDX84	9.477	60,427,976	52,687,712 (87.19%)	94.99	38.95
Yunnan	YX11	12.699	84,662,326	72,355,540 (91.53%)	93.435	38.645
	YX17	12.647	84,314,934	71,056,978 (90.59%)	93.265	38.61
	YX22	12.896	85,972,880	71,176,766 (89.7%)	92.725	38.505
	YX26	11.978	79,852,688	66,846,012 (90.59%)	92.915	38.54
Hunan	JY1	15.441	67,826,812	58,578,180 (86.36%)	94.575	38.825
	JY2	12.671	81,534,830	73,602,632 (90.27%)	95.32	39.035
	JY3	13.696	88,105,784	77,482,064 (87.94%)	95.12	38.99
	JY4	26.533	163,795,202	31,242,094 (19.07%)	93.61	38.69

**Table 2:** Viral infection characteristics in wild citrus samples from different regions. The table compares the number of virus species and mixed infection rates in Yunnan, Jiangxi, and Hunan.

Region	Total Sample	Number of Virus Species	Number of Mixed Infection Samples (Number of Samples with ≥2 Viruses)
Yunnan	4	4(CEVd, CTV, CAAV1, CiVB)	4
Jiangxi	3	2(CEVd, CTV)	3
Hunan	4	2(CEVd, CTV)	4

## Data Availability

The original contributions presented in this study are included in the article/Supplementary Material. Further inquiries can be directed at the corresponding authors.

## Supplementary Materials

The following supporting information can be downloaded at: https://www.mdpi.com/article/10.3390/v17091162/s1: Table S1: Primers used in this study. Table S2: The sequences used for Bayesian phylogenetic analysis in this study. Table S3: AMOVA for the CTV near-full-length genome sequences.
