# Supplementary material for: Wild Citrus CTV Genomic Data Provides Novel Insights into Its Global Transmission Dynamics
Source: Viruses. 2025 Aug 26;17(9):1162. doi: 10.3390/v17091162 (PMC12474035; doi:10.3390/v17091162)
Supplement: Supplementary file 1 [file viruses-17-01162-s001.zip › Supplementary Information1.pdf]

Table S1:Primers used in this study

Viruses	Primer names	Primer sequence (5'-3')
Citrus tristeza virus	CP-F	CTGCTTTAAGGGTCGTTAATTG
	CP-R	TGAAACTCCACCATCCCGAT
Citrus associated ampelovirus 1	CAV1_df	CGATGTATTAGGGTGACGACTC
	CAV1_dr	TCACTGTAAACTTTCGCTCCTG
Citrus virus B	CVB_df	AATTGTGCCTGTTATAGCTGGA
	CVB_dr	AGCTGTGTTCTCATAGCCTTGT
Citrus Exocortis Viroid	CEV-F	GGAAACCTGGAGGAAGTCGAG
	CEv-R	CCGGGGATCCCTGAAGGACTT
