# Supplementary material for: Wild Citrus CTV Genomic Data Provides Novel Insights into Its Global Transmission Dynamics
Source: Viruses. 2025 Aug 26;17(9):1162. doi: 10.3390/v17091162 (PMC12474035; doi:10.3390/v17091162)
Supplement: Supplementary file 1 [file viruses-17-01162-s001.zip › Supplementary Information2.pdf]

Table S2: The sequences used in this study

No.	Host Origin	Country	Date	Accessions.NO	name
CTV001	Citrus sinensis	Angola	2017	MW388809.1	MW388809/CTV-AO5
CTV002	Citrus sinensis	Brazil	2007	KY110737.1	KY110737/CTV-strain CSL01
CTV003	Citrus sinensis	Brazil	2007	KY110738.1	KY110738/CTV-CSL02
CTV004	Citrus	China	2019	MZ672134.1	MZ672134/CTV-2019-30-2
CTV005	Citrus	China	2019	MZ672136.1	MZ672136/CTV-2019-7-DY
CTV006		China	2017	MH558665.1	MH558665/CTV-CN-RB-9
CTV007		China	2017	MH323442.1	MH323442/CTV-CN-M1-ZT1
CTV008	Citrus sinensis	China	2005	MK779711.1	MK779711/CTV-N4
CTV009	Citrus sinensis	China	2008	OQ708956.1	OQ708956/CTV-S45-1
CTV010	Citrus sinensis	China	2008	OQ708957.1	OQ708957/CTV-S45-2
CTV011	Citrus reticulata	China	2018	MW365403.1	MW365403/CTV-YN1-6
CTV012	Citrus sinensis	China	2006	JQ911664.1	JQ911664/CTV-CT11A
CTV013	Citrus	China	2019	MZ672135.1	MZ672135/CTV-2019-30-3
CTV014	Citrus	China	2019	MZ692538.1	MZ692538/CTV-2019-6-DY
CTV015	Citrus reticulata	China	2017	MK491895.1	MK491895/CTV-FN08
CTV016	Citrus sinensis	China	2007	KU720382.1	KU720382/CTV-HU-PSTS
CTV017	Citrus sinensis	China	2011	JQ061137.1	JQ061137/CTV-AT-1
CTV018		China	2017	MH558666.1	MH558666/CTV-CN-RB-L13
CTV019	Citrus reticulata	China	2016	MH593380.1	MH593380/CTV-CT91-A1
CTV020	Citrus sinensis	China	2006	JQ911663.1	JQ911663/CTV-CT14A
CTV021		China	2017	MH323441.1	MH323441/CTV-CN-L1-ZT1
CTV022	Citrus daoxianensis	China	2019	ON094625.1	ON094625/CTV-JY-2
CTV023	Citrus reticulata	China	2019		JY1
CTV024	Citrus reticulata	China	2019		JY2
CTV025	Citrus reticulata	China	2019		JY3
CTV026	Citrus reticulata	China	2019		JY4
CTV027	Citrus reticulata	China	2024		NDX45
CTV028	Citrus reticulata	China	2024		NDX50
CTV029	Citrus reticulata	China	2024		YX11
CTV030	Citrus reticulata	China	2024		YX17
CTV031	Citrus reticulata	China	2024		YX26
CTV032	Citrus sinensis	Croatia	2022	OR184846.1	OR184846/CTV-9A
CTV033		Ecuador	2018	MZ870354.1	MZ870354/CTV-RB
CTV034		France	2020	MZ648331.1	MZ648331/CTV-C7B1
CTV035	Citrus sinensis	Greece	2022	OR225235.1	OR225235/CTV-O2319Gr
CTV036	Citrus sinensis	Greece	2010	KF908013.1	KF908013/CTV-Crete 1825
CTV037	Citrus reticulata	India	2002	HM573451.1	HM573451/CTV-Kpg 3
CTV038	Citrus sinensis	Iran	2016	OR192038.1	OR192038/CTV-IR-North2
CTV039	Citrus	Iran	2022	OP900953.1	OP900953/CTV-Sari
CTV040	Citrus sinensis	Iran	2016	OR192037.1	OR192037/CTV-IR-North1
CTV041	Citrus x aurantiifolia	Iran	2016	OR192039.1	OR192039/CTV-IR-South1

CTV042	Citrus x limon	Iran	2016	OR192040.1	OR192040/CTV-IR-South2
CTV043	Citrus macrophylla	Italy	2014	KR263170.1	KR263170/CTV-Mac25
CTV044	Citrus sinensis	Italy	2007	KC748391.1	KC748391/CTV-Bau282
CTV045	Citrus macrophylla	Italy	2012	KJ790175.1	KJ790175/CTV-Mac39
CTV046	Citrus sinensis	Italy	2007	KC748392.1	KC748392/CTV-SG29
CTV047		Montenegro	2022	OR147839.1	OR147839/ CTV-168/22-2
CTV048		Montenegro	2018	OR122725.1	OR122725/ CTV-167/18
CTV049	Citrus sinensis	Montenegro	2022	OR122726.1	OR122726/ CTV-127/22
CTV050	Citrus reticulata	Montenegro	2018	OP006457.1	OP006457/CTV- 186/18-1
CTV051	Citrus sinensis	Montenegro	2022	OR147840.1	OR147840/ CTV-170/22
CTV052	Citrus limon	Montenegro	2020	OP006456.1	OP006456/ CTV-60/20
CTV053	Citrus sinensis	Montenegro	2022	OR122730.1	OR122730/ CTV-166/22
CTV054	Citrus sinensis	Montenegro	2022	OR147841.1	OR147841/ CTV-125/22-1
CTV055	Citrus sinensis	Montenegro	2022	OR147842.1	OR147842/ 1CTV-25/22-2
CTV056		Montenegro	2022	OR147838.1	OR147838/ CTV-168/22-1
CTV057	Citrus reticulata	Montenegro	2018	OP006458.1	OP006458/ CTV-186/18-2
CTV058		Montenegro	2022	OR122728.1	OR122728/ CTV-151/22-2
CTV059		Montenegro	2022	OR122729.1	OR122729/ CTV-151/22-3
CTV060		Montenegro	2022	OR122727.1	OR122727/ CTV-151/22-1
CTV061	Citrus trifoliata	New Zealand	2005	FJ525435.1	FJ525435/CTV-NZRB-M17
CTV062	Citrus trifoliata	New Zealand	2005	FJ525434.1	FJ525434/CTV-NZRB-TH30
CTV063	Citrus trifoliata	New Zealand	2005	FJ525431.1	FJ525431/CTV-NZRB-M12
CTV064	Citrus trifoliata	New Zealand	2005	FJ525432.1	FJ525432/CTV-NZRB-G90
CTV065	Citrus trifoliata	New Zealand	2005	FJ525433.1	FJ525433/CTV-NZRB-TH28
CTV066	Citrus sinensis	New Zealand	2005	FJ525436.1	FJ525436/CTV-NZ-B18
CTV067	Citrus aurantiifolia	New Zealand|	2006	EU857538.1	EU857538/CTV-SP
CTV068	Citrus sinensis	Puerto Rico	1992	JF957196.1	JF957196/CTV-B301
CTV069	Citrus aurantiifolia	South Africa	1996	KU883267.1	KU883267/CTV-LMS6-6
CTV070	Citrus aurantiifolia	South Africa	2000	KU883265.1	KU883265/CTV-B390-5
CTV071	Citrus aurantiifolia	South Africa	2000	MH051717.1	MH051717/CTV-B389-1
CTV072	Citrus aurantiifolia	South Africa	2000	MH051718.1	MH051718/CTV-B389-4
CTV073	Citrus aurantiifolia	South Africa	2015	MH051719.1	MH051719/CTV-T3-KB
CTV074	Citrus aurantiifolia	South Africa	2011	KU883266.1	KU883266/CTV-Maxi
CTV075	Citrus aurantiifolia	South Africa	1996	MT350595.1	MT350595/CTV-T68 GFMS12-7
CTV076	Citrus aurantiifolia	South Africa	1996	MT350596.1	MT350596/CTV-T68 GFMS12-9
CTV077	Citrus x paradisi	South Africa	2004	MT350597.1	MT350597/CTV-T68 GFMS12_Duncan
CTV078	Citrus aurantiifolia	South Africa	2017	MK033510.2	MK033510/CTV-GFMS12-1.3
CTV079	Citrus aurantiifolia	South Africa	1996	MK033511.2	MK033511/CTV-GFMS12-8
CTV080	Citrus aurantiifolia	South Africa	2012	KC333868.1	KC333868/CTV-CT-ZA3
CTV081	Citrus aurantiifolia	South Africa	2012	KC333869.1	KC333869/CTV-CT-ZA2
CTV082	Citrus unshiu	South Korea	2022	LC775311.1	LC775311/CTV-CNU-ONT RNA
CTV083	Citrus sinensis	Spain	2023	OQ848758.1	OQ848758/CTV-141.5 genotype RB
CTV084	Citrus sinensis	Spain	2023	PQ538530.1	PQ538530/CTV-IVIA141.4.RB2
CTV085	Citrus x clementina	Spain	2023	PQ576739.1	PQ576739CTV-/1423 T30

CTV086	Citrus x clementina	Spain	2023	PQ576744.1	PQ576744/CTV-152.7 T30
CTV087	Citrus sinensis	Spain	2023	PQ576737.1	PQ576737/CTV-141.5 T30
CTV088	Citrus x clementina	Spain	2023	PQ538529.1	PQ538529/CTV-IVIA1423.RB1
CTV089	Citrus sinensis	Spain	2023	PQ576738.1	PQ576738/CTV-141.5 VT
CTV090	Citrus x clementina	Spain	2023	PQ576740.1	PQ576740/CTV-1423 VT
CTV091	Citrus x clementina	Spain	2023	PQ576741.1	PQ576741/CTV-152.7 VT
CTV092	Citrus sinensis	Spain	2023	PQ576743.1	PQ576743/CTV-141.4 VT
CTV093	Citrus sinensis	Spain	2023	OQ714508.1	OQ714508/CTV-137.4
CTV094	Citrus sinensis	Spain	2023	PQ576736.1	PQ576736/CTV-137.4 T30
CTV095	Citrus sinensis	Spain	2023	PQ576742.1	PQ576742/CTV-141.4 T30
CTV096	Citrus reticulata	Thailand	2010	JQ798289.1	JQ798289/CTV-A18
CTV097	Citrus sinensis	Uruguay	2014	MH186146.1	MH186146/CTV-DSST-17
CTV098	Citrus trifoliata	Uruguay	2015	OP448604.1	OP448604/CTV-RB-UY1
CTV099	Citrus macrophylla	USA	2013	MK018120.1	MK018120/CTV-1479
CTV100	Citrus sinensis	USA	2004	EU937521.1	EU937521/CTV-strain T36
CTV101	Citrus aurantium	USA	2014	MH279617.1	MH279617/CTV-CCTEA96339
CTV102	Citrus sinensis	USA	2012	KC517485.1	KC517485/CTV-FS674-T36
CTV103	Citrus reticulata	USA	2012	KC517486.1	KC517486/CTV-FS701-T36
CTV104	Citrus macrophylla	USA	2012	KC517488.1	KC517488/CTV-FS577
CTV105	Citrus reticulata	USA	2012	KC517487.1	KC517487/CTV-FS703-T36
CTV106	Citrus reticulata	USA	2010	KU358530.1	KU358530/CTV-CA-RB-AT35
CTV107	Citrus x latifolia	USA	2001	GQ454870.1	GQ454870/CTV-HA16-5
CTV108	Citrus sinensis	USA	2004	EU937520.1	EU937520/CTV-T30
CTV109	Citrus sinensis	USA	2022	PQ603092.1	PQ603092/CTV-T30 Woodlake 1
CTV110	Citrus	USA	2014	MH279618.1	MH279618/CTV-702 5a
CTV111	Citrus reticulata	USA	2010	KU578007.1	KU578007/CTV-CA-T30-AT4
CTV112	Citrus reticulata	USA	2012	KC517489.1	KC517489/CTV-FS701-T30
CTV113	Citrus sinensis	USA	2002	KU361340.1	KU361340/CTV-CA-RB-115
CTV114	Citrus reticulata	USA	2010	KU356770.1	KU356770/CTV-CA-RB-AT25
CTV115	Citrus macrophylla	USA	2005	KC525952.1	KC525952/CTV-T3
CTV116	Citrus reticulata	USA	2012	KC517491.1	KC517491/CTV-FS703-T30
CTV117	Citrus reticulata	USA	2010	KU361339.1	KU361339/CTV-CA-VT-AT39
CTV118	Citrus sinensis	USA	2004	EU937519.1	EU937519/CTV-VT
CTV119	Citrus x paradisi	USA	2012	KC517490.1	KC517490/CTV-FL278-T30
CTV120	Citrus sinensis	USA	2012	KC517493.1	KC517493/CTV-FL202-VT
CTV121	Citrus reticulata	USA	2012	KC517494.1	KC517494/CTV-FS701-VT
CTV122	Citrus medica	USA	2010	KU589212.1	KU589212/CTV-CA-S1-L
CTV123	Citrus sinensis	USA	2001	KU589213.1	KU589213/CTV-CA-S1-L65
CTV124	Citrus macrophylla	USA	2003	JQ965169.1	JQ965169/CTV-T68-1
CTV125	Citrus x latifolia	USA	2001	GQ454869.1	GQ454869/CTV-HA18-9
CTV126	Citrus reticulata	USA	2012	KC517492.1	KC517492/CTV-FS703-VT
