# Supplementary material for: Wild Citrus CTV Genomic Data Provides Novel Insights into Its Global Transmission Dynamics
Source: Viruses. 2025 Aug 26;17(9):1162. doi: 10.3390/v17091162 (PMC12474035; doi:10.3390/v17091162)
Supplement: Supplementary file 1 [file viruses-17-01162-s001.zip › Supplementary Information3.pdf]

Table S3. AMOVA for the CTV near-full-length genome sequences.

Source of variation	d.f	Sum of squares	Variance components	Percentage of variation
Among populations	5	10144.197	69.45195	9.28%
Within populations	120	81485.549	679.04624	90.72%
GeneFlow	$N_{m:}$ 2.44			
